# Intravaginal *Chlamydia trachomatis* Challenge Infection Elicits T_H_1 and T_H_17 Immune Responses in Mice That Promote Pathogen Clearance and Genital Tract Damage

**DOI:** 10.1371/journal.pone.0162445

**Published:** 2016-09-08

**Authors:** Rodolfo D. Vicetti Miguel, Nirk E. Quispe Calla, Stephen D. Pavelko, Thomas L. Cherpes

**Affiliations:** 1 Department of Microbial infection & Immunity, The Ohio State University College of Medicine, Columbus, Ohio, United States of America; 2 Department of Obstetrics & Gynecology, The Ohio State University College of Medicine, Columbus, Ohio, United States of America; Xavier Bichat Medical School, INSERM-CNRS - Université Paris Diderot, FRANCE

## Abstract

While ascension of *Chlamydia trachomatis* into the upper genital tract of women can cause pelvic inflammatory disease and Fallopian tube damage, most infections elicit no symptoms or overt upper genital tract pathology. Consistent with this asymptomatic clinical presentation, genital *C*. *trachomatis* infection of women generates robust T_H_2 immunity. As an animal model that modeled this response would be invaluable for delineating bacterial pathogenesis and human host defenses, herein we explored if pathogen-specific T_H_2 immunity is similarly elicited by intravaginal (ivag) infection of mice with oculogenital *C*. *trachomatis* serovars. Analogous to clinical infection, ascension of primary *C*. *trachomatis* infection into the mouse upper genital tract produced no obvious tissue damage. Clearance of ivag challenge infection was mediated by interferon (IFN)-γ-producing CD4^+^ T cells, while IFN-γ signaling blockade concomitant with a single ivag challenge promoted tissue damage by enhancing *Chlamydia*-specific T_H_17 immunity. Likewise, IFN-γ and IL-17 signaling blockade or CD4^+^ T cell depletion eliminated the genital pathology produced in untreated controls by multiple ivag challenge infections. Conversely, we were unable to detect formation of pathogen-specific T_H_2 immunity in *C*. *trachomatis*-infected mice. Together, our work revealed *C*. *trachomatis* infection of mice generates T_H_1 and T_H_17 immune responses that promote pathogen clearance and immunopathological tissue damage. Absence of *Chlamydia*-specific T_H_2 immunity in these mice newly highlights the need to identify experimental models of *C*. *trachomatis* genital infection that more closely recapitulate the human host response.

## Introduction

*Chlamydia trachomatis*, the most common sexually transmitted bacterium, is exclusively a pathogen of humans [1]. Though genital *C*. *trachomatis* infection in women may cause pelvic inflammatory disease (PID) and Fallopian tube damage that increases the chances for ectopic pregnancy and infertility [2, 3], the vast majority of infections are asymptomatic and produce no adverse reproductive outcomes [4]. Likewise, *C*. *trachomatis* often resides in the human female genital tract for months without inducing any overt inflammatory changes [4]. While numerous countries have implemented public health programs to heighten identification and treatment of individuals with asymptomatic genital *C*. *trachomatis* infection, most did not achieve sustainable decreases in population disease prevalence [5]. These findings indicate that vaccination may be the prevention strategy more likely to reduce sexual *C*. *trachomatis* transmission, but no vaccine designed to protect against genital chlamydial infection has been evaluated clinically.

The best accepted and explored strategy for developing such a vaccine is based on results from mouse models of genital *Chlamydia muridarum* infection in which protection against secondary infection is conferred by *Chlamydia*-specific T_H_1-type immunity [6, 7]. A strength of this model is that infected mice develop hydrosalpinx that resembles the Fallopian tube pathology seen in a small subset of *C*. *trachomatis*-infected women. As with most experimental infections however, limitations associated with this infection model reduce its ability to recapitulate clinical disease. Specifically, *C*. *muridarum* colonizes the mouse cecum but does not naturally infect the female genital tract [8]. In addition, genomic analysis of *C*. *muridarum* and *C*. *trachomatis* show an early separation in their evolutionary histories [9]. There are also important dissimilarities in disease pathogenesis, as only *C*. *muridarum* escapes IFN-γ-mediated immunity by blocking the effects of IRG proteins [10]. *C*. *muridarum* replication is also more rapid, and its systemic dissemination more closely resembles the infection course associated with *C*. *trachomatis* lymphogranuloma venereum (LGV) serovars than the more epithelium-restricted infections associated with oculogenital serovars [11]. Congruent with greater tissue invasiveness, genital infection of mice with *C*. *muridarum* or *C*. *trachomatis* LGV serovars elicits exuberant T_H_1-mediated inflammation that rapidly eradicates infection and produces extensive upper genital tract (UGT) damage [6, 7, 10]. In comparison, genital infection of women with an oculogenital *C*. *trachomatis* serovar less frequently induces overt inflammation [3, 4], and generates prominent pathogen-specific T_H_2 immunity [12–14]. As these differences in phylogeny, natural infection, pathogenesis, inflammation, and adaptive immunity indicate that genital infection of mice with *C*. *muridarum* does not fully model clinical disease, herein we sought to delineate murine host responses to intravaginal (ivag) primary and challenge infection with an oculogenital *C*. *trachomatis* serovar. Specifically, we explored if ivag *C*. *trachomatis* infection induces the formation of T_H_2 immunity.

## Results

### Primary infection of mice with an oculogenital *C*. *trachomatis* serovar ascended from the lower to upper genital tract

Phenotypic disease expression in women with genital *C*. *trachomatis* infection is rare, but can have devastating consequences for reproductive fertility [15]. Most studies that used mouse models to explore chlamydial pathogenesis used *C*. *muridarum* rather than *C*. *trachomatis*, as the former was thought to have greater capacity to migrate into UGT tissue and cause oviduct damage [7, 11, 16]. It was also thought ivag infection of mice with oculogenital *C*. *trachomatis* serovars induces minimal endometrial inflammation and UGT pathology, and that ascension of ivag *C*. *trachomatis* infection beyond the cervix of mice requires large infectious inoculums (i.e., 10^6^–2 x 10^7^ inclusion-forming units (IFU) of the bacterium) [17–21]. We therefore began our investigation of the murine response to an oculogenital *C*. *trachomatis* serovar by characterizing primary ivag infection in Balb/cJ mice that were infected daily for 3 successive days with 10^4^ IFU of *C*. *trachomatis* serovar D. This inoculum approximates the levels detected in human genital secretions [22], and is 100- to 2000-fold lower than doses previously explored [17–21].

**Fig 1:**
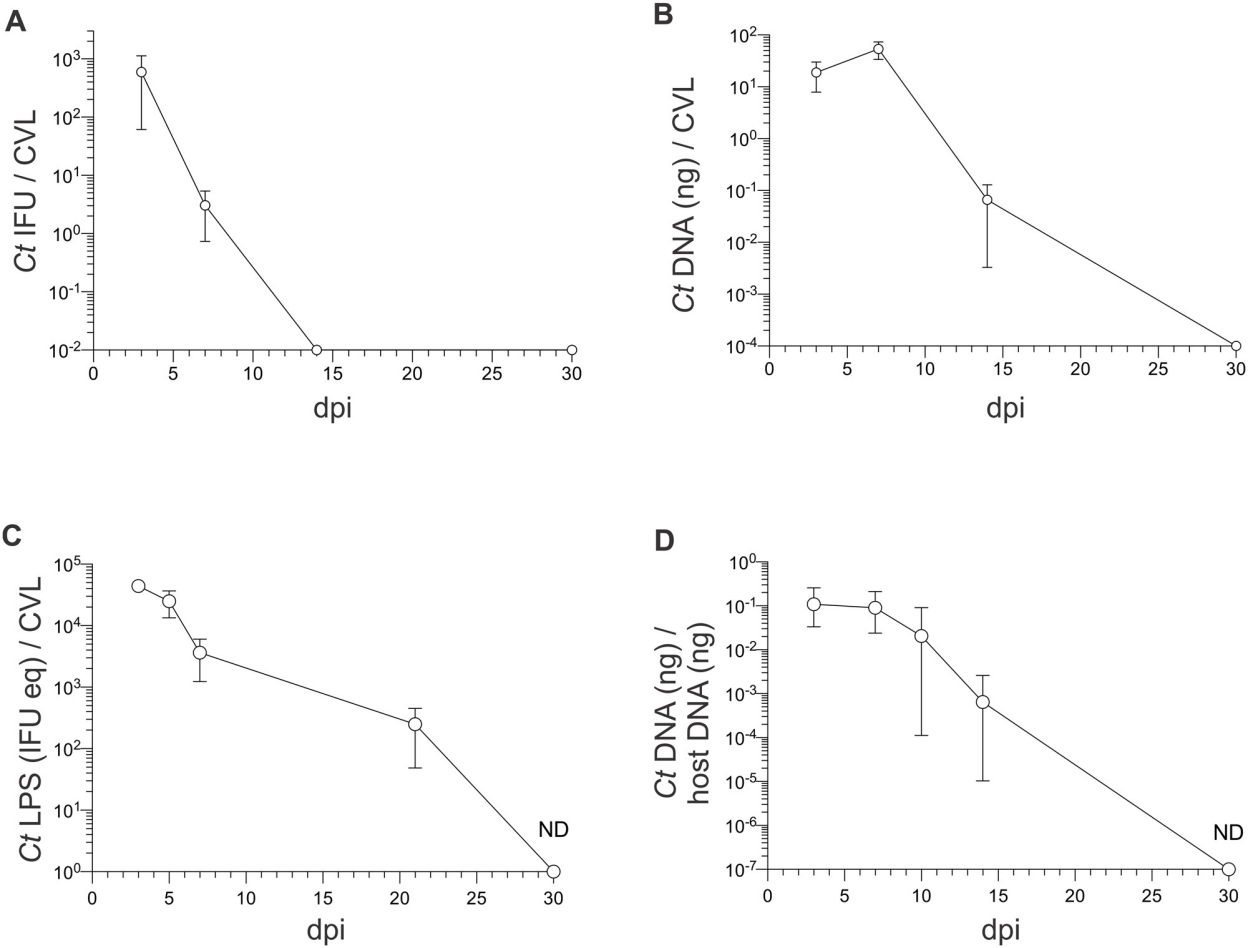
Intravaginal (ivag) *C*. *trachomatis* infection of mice ascends to the upper genital tract (UGT). 6–8 week old female Balb/cJ mice were injected s.c. with 1 mg DMPA 5 days prior to ivag infection with 10^4^ IFU of *C*. *trachomatis* (*Ct*) serovar D (mice were infected daily for 3 consecutive days). On indicated dpi, cervicovaginal lavage (CVL) specimens were collected to assess *Chlamydia* clearance via (A) IFU assays (B) RT-qPCR that measured *Chlamydia* DNA levels and (C) ELISA that quantitated C*hlamydia* lipopolysaccharide (LPS) levels; (mean ±SD, n = 5 per group). (D) in separate studies, mice were euthanized on specified dpi, UGT excised, and *Chlamydia* DNA and host DNA quantified via RT-qPCR; (median ± range, n = 5 per time point) (ND, non-detectable) (displayed results representative of 2–3 independent experiments).

**Fig 2:**
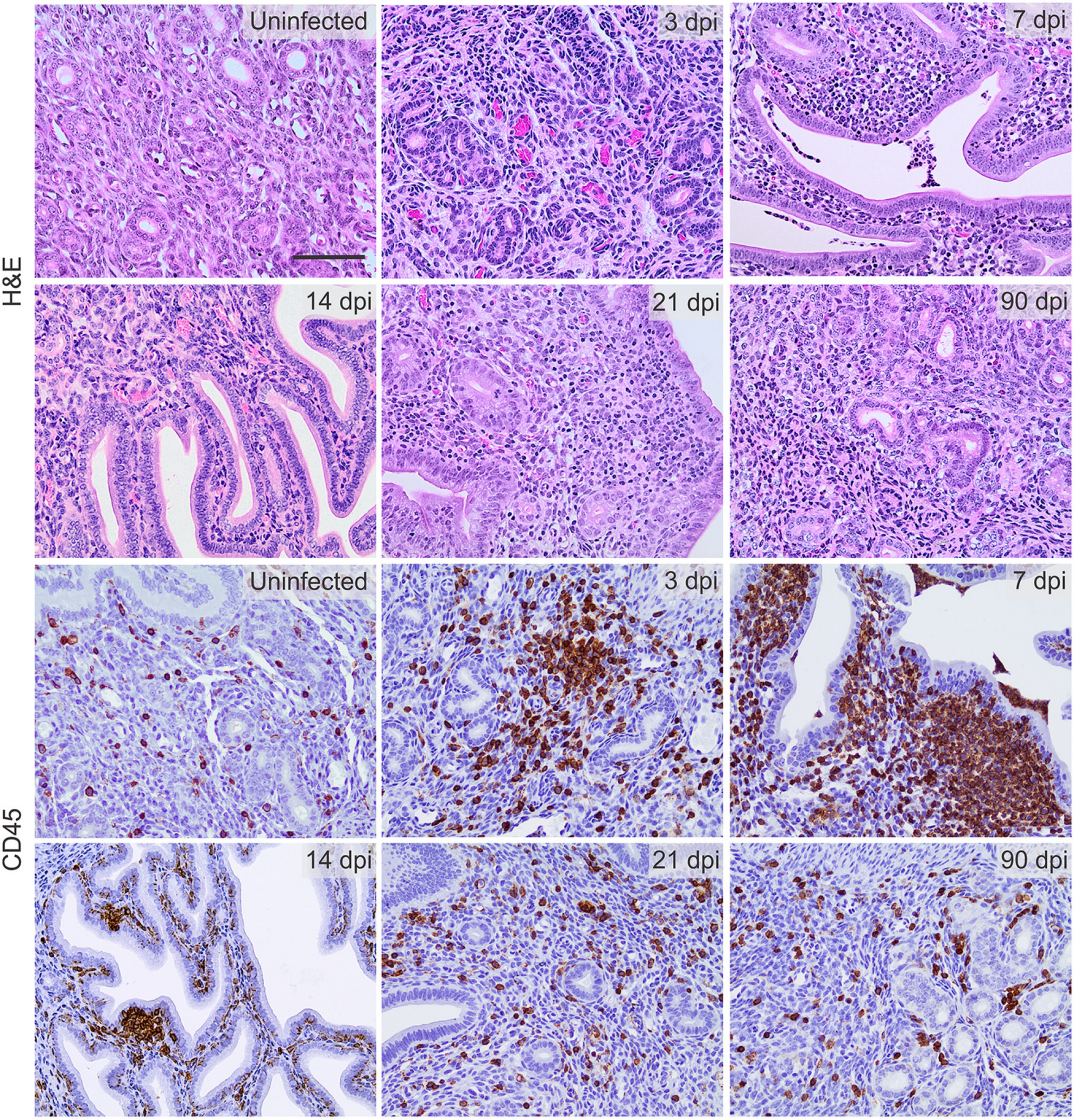
Primary ivag *C*. *trachomatis* infection elicits robust endometrial inflammation. As denoted in Fig 1, Balb/cJ mice were infected with 10^4^ IFU of *C*. *trachomatis* serovar D. Animals were euthanized at indicated dpi, and UGT tissue used to characterize inflammation by H&E staining (upper panels) or IHC of CD45^+^ cell infiltrates; (lower panels) (n = 5–10 per group). Representative images indicate endometrial leukocyte infiltration was particularly intense at 7 dpi; (scale bar, 50 μm) (displayed results are representative of 2–3 independent experiments).

We collected cervicovaginal lavages (CVL) at various days post infection (dpi) to quantify levels of infectious *C*. *trachomatis* elementary bodies (EB), chlamydial DNA and lipopolysaccharide (LPS), and these studies revealed peak *C*. *trachomatis* levels were present in CVL specimens obtained 3–7 dpi (Fig 1A–1C). Interestingly, chlamydial DNA was also detected in all UGT tissues collected at 14 dpi (Fig 1D), and we found a strong positive correlation between UGT bacterial burden and endometrial inflammation intensity (Fig 2). Conversely, at time points after primary infection had been resolved (e.g., 90 dpi), mice showed levels of endometrial inflammation and UGT pathology comparable to those seen in uninfected controls (S1 Fig). At similar time points after primary infection, we also saw no discernable damage in the UGT of mice infected with 10^4^ IFU of *C*. *trachomatis* serovar E or *C*. *trachomatis* LGV serovar L2 (S1 Fig). Comparable to earlier reports [17–19], we also found that primary ivag infection with a single 10^6^ IFU inoculum of *C*. *trachomatis* serovar D (either ivag or transcervically) caused no obvious UGT damage in mice examined at 90 dpi (S1 Fig). On the other hand, primary ivag *C*. *muridarum* infection of Balb/cJ mice induced oviduct damage and hydrosalpinx formation in the majority of infected mice (S1 Fig). Of note, this UGT pathology mirrored the pathology we detected in IFN-γ^-/-^ mice after primary genital infection with *C*. *trachomatis* serovar D (S2 Fig). Together, our initial studies established primary ivag infection of wild type Balb/cJ mice with 10^4^ IFU of an oculogenital *C*. *trachomatis* serovar consistently caused an ascending infection that was eradicated without producing the extensive UGT tissue destruction elicited by ivag infection with *C*. *muridarum*.

### CD4^+^ T cells controlled *C*. *trachomatis* challenge infection

As primary ivag infection with oculogenital *C*. *trachomatis* serovars caused productive infection that was eradicated by 30 dpi, we next explored *C*. *trachomatis*-induced T cell responses by intravaginally challenging mice. This strategy was selected based on earlier studies that showed more robust T cell responses and strongest protection from challenge infection in mice that had been primarily infected with live *C*. *muridarum* EB [23, 24]. Prior studies also showed protection against *C*. *muridarum* challenge infection was more dependent on CD4^+^ vs. CD8^+^ T cells [25, 26], and that CD4^+^ T cells conferred protection if *C*. *trachomatis* LGV serovars were inoculated directly into the mouse uterus [20]. In consideration of these studies, we posited that CD4^+^ T cells similarly control ivag challenge infection with oculogenital *C*. *trachomatis* serovars.

**Fig 3:**
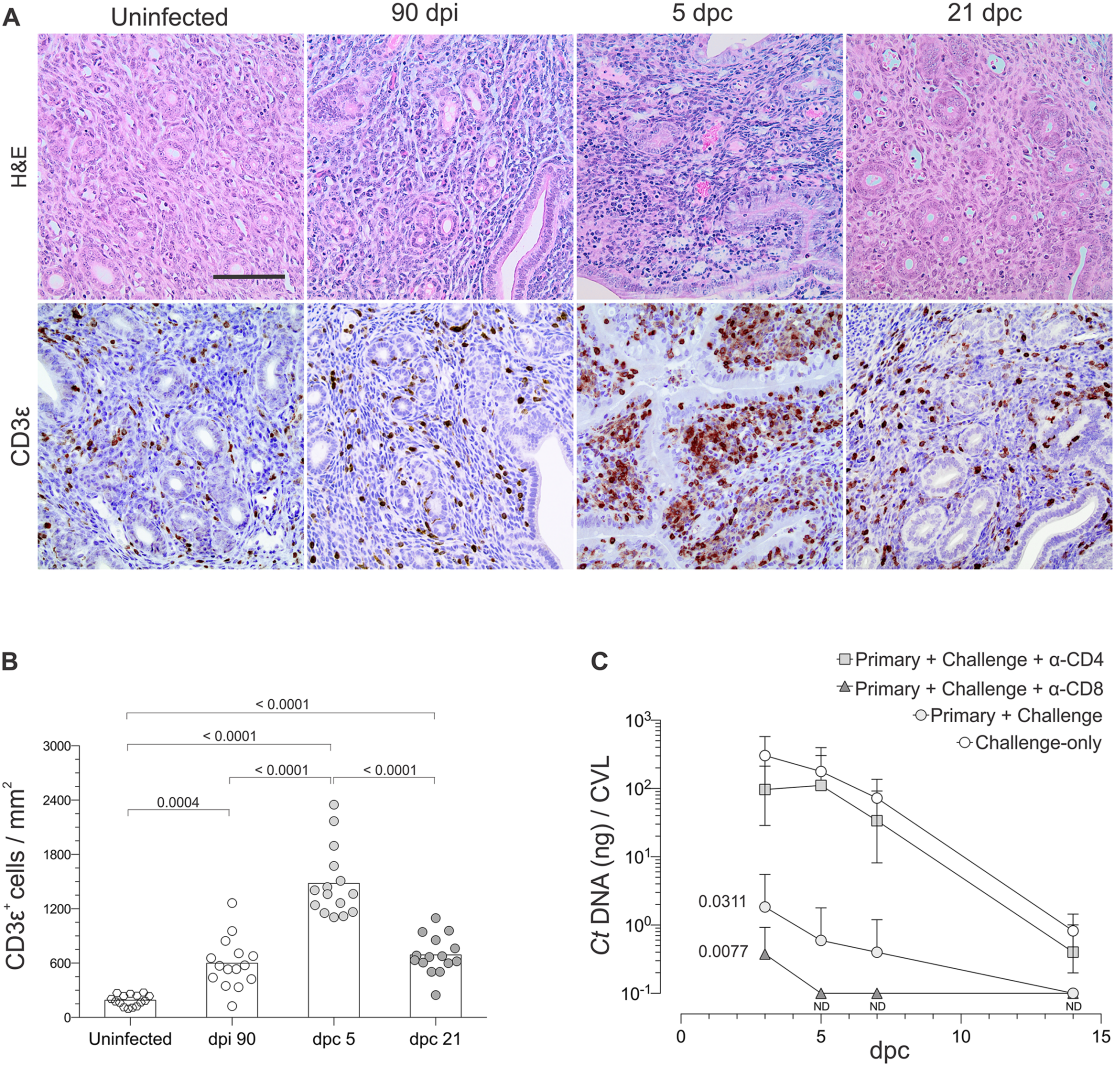
CD4^+^ T cells controlled ivag *C*. *trachomatis* challenge infection. Uninfected Balb/cJ mice were ivag infected with *C*. *trachomatis* as described in Fig 1 or remained uninfected. (A) Uninfected mice or mice 90 dpi were ivag challenged with 10^6^ IFU of *C*. *trachomatis* serovar D. At indicated dpi or dpc, mice were euthanized and UGT processed for H&E staining or analysis of CD3ε^+^ cells by IHC; representative images reveal intense mononuclear CD3ε^+^ infiltrate at 5 dpc (scale bar, 50 μm). (B) Quantification of CD3ε^+^ cell infiltrates with ImageJ software (as described in Methods) showed no statistically significant differences between mice at dpi 90 and dpc 21 (n = 15 per group) (bars indicate means). (C) *Chlamydia* DNA levels were determined by RT-qPCR mice in CVL specimens collected from mice during primary ivag infection, after ivag challenge, and after ivag challenge of mice administered antibodies depleting CD4^+^ or CD8^+^ T cells 1 day prior to challenge and every other day until euthanasia. Areas under the curve (AUC) for bacterial clearance were compared as described in Methods, and this revealed CD4^+^ T cells were the T cell subset that controlled *Chlamydia* challenge; (n = 5 per group) (values are mean ±SD) (results representative of 2 independent experiments) (ND, non-detectable).

Initial histological examination of genital tracts from uninfected mice and mice 21 days post-challenge (dpc) showed a single ivag challenge caused no discernable increase in genital pathology. IHC analyses of endometrial tissue sections from uninfected mice, or mice 90 dpi, 5 dpc, or 21 dpc demonstrated the more robust T cell response occurred at 5 dpc (Fig 3A and 3B). Based on this exuberant endometrial response, we explored the role of T cells in controlling *C*. *trachomatis* by comparing chlamydial DNA levels in CVL obtained from mice during primary ivag infection, after ivag challenge infection, or after ivag challenge infection in which mice received antibodies depleting CD4^+^ or CD8^+^ cells concomitant with challenge. In these studies, we found *C*. *trachomatis* challenge infection was eradicated significantly faster than primary infection, and that CD4^+^ T cells were chiefly responsible for this enhanced clearance (Fig 3C).

### *C*. *trachomatis*-specific CD4^+^ T cells displayed T_H_1 and T_H_17 effector function

**Fig 4:**
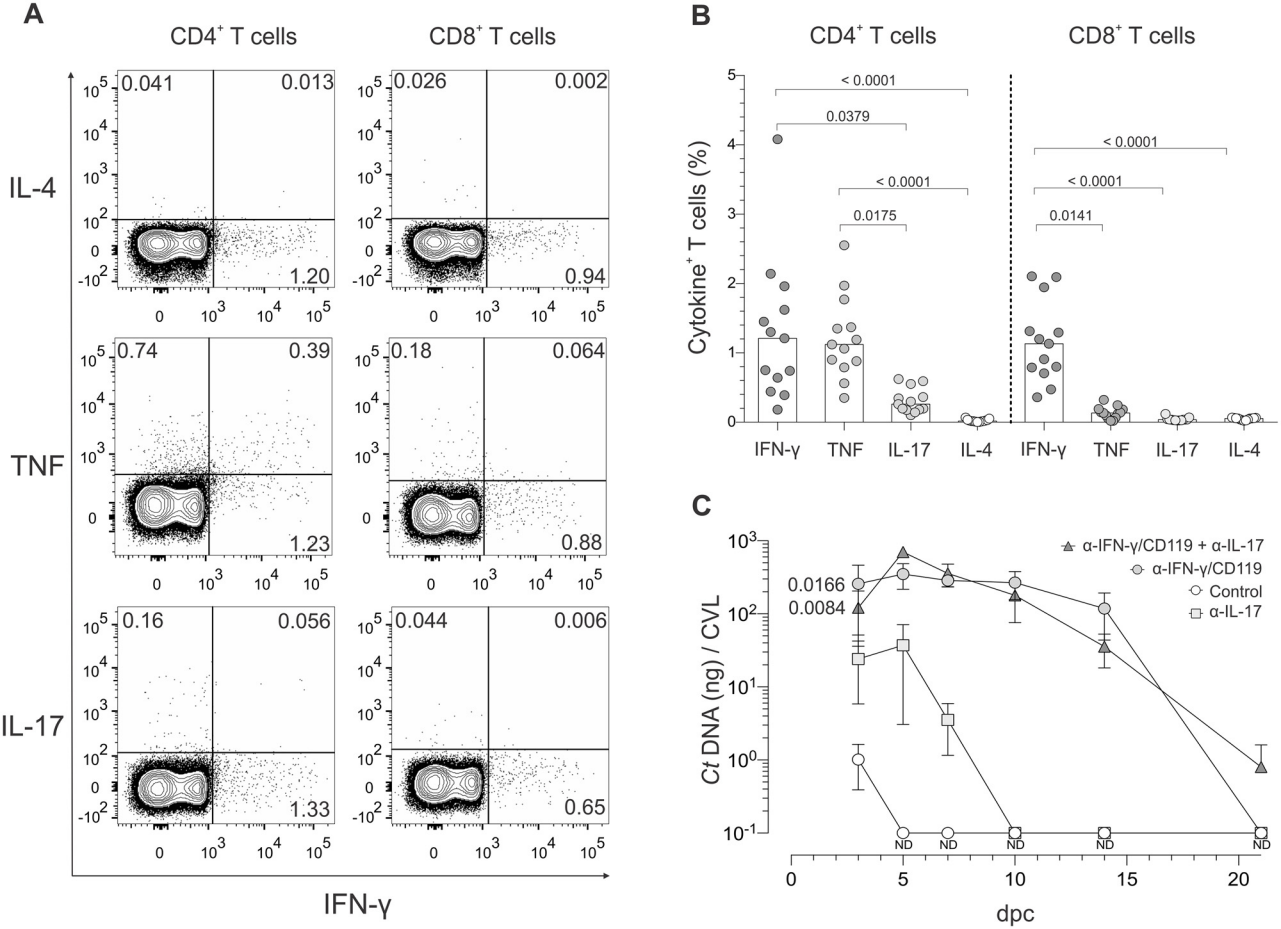
*C*. *trachomatis*-specific CD4^+^ T cells displayed T_H_1 and T_H_17 effector function, but control of ivag *C*. *trachomatis* challenge is mainly mediated by T_H_1 immunity. At 60 dpi, mice were ivag challenged with 10^6^ IFU of *C*. *trachomatis* serovar D. At 5 dpc, animals were euthanized, DLN excised and processed into single-cell suspensions, and cells incubated with inactivated *Chlamydia* EB or media alone. (A) Representative contour plots from the intracellular cytokine staining (ICS) assay used to quantify secretion of IFN-γ, TNF, IL-17, and IL-4 by CD4^+^ and CD8^+^ T cells that responded to stimulation with *Chlamydia* EB; (quadrant numbers indicate percentages of cytokine-producing cells). (B) Percentages of cytokine-producing CD4^+^ and CD8^+^ T cells; (n = 13) (bars indicate medians). (C) Using additional Balb/cJ mice at 60 dpi, 10^6^ IFU of *C*. *trachomatis* serovar D was ivag administered to untreated controls or mice treated with antibodies blocking IFN-γ, IL-17, or IFN-γ and IL-17 signaling concomitant with challenge. At various days after challenge, CVL specimens were collected to measure *Chlamydia* DNA levels via RT-qPCR. AUC analysis for bacterial clearance revealed that *Chlamydia* challenge was primarily controlled by T_H_1 immunity; (n = 5 per group) (values are means ±SD) (results representative of 2 independently performed studies) (ND, non-detectable).

As mice were protected against ivag challenge infection by CD4^+^ T cells, we sought to define T cell responses in these animals. For this, iliac lymph nodes were excised from Balb/cJ mice at 5 dpc, processed into single-cell suspensions, and stimulated with inactivated *C*. *trachomatis* serovar D EB. We then used a flow-cytometry-based intracellular cytokine staining (ICS) assay to delineate T cell effector function (Fig 4A). In contrast to the robust and durable T_H_2 immunity seen in women with genital *C*. *trachomatis* infection [12], our data analysis revealed *Chlamydia*-specific CD4^+^ and CD8^+^ T cell effector functions characterized primarily by IFN-γ production, lesser amounts of IL-17, and no appreciable intracellular accumulation of IL-4 (Fig 4B). While CD4^+^ T cells also secreted TNF in response to *ex vivo* EB stimulation, no comparable response was detected in CD8^+^ T cells (Fig 4B). Performing identical studies with iliac lymph nodes from C57BL/6J mice, we observed pathogen-specific T cell effector functions that closely resembled those seen in *C*. *trachomatis*-infected Balb/cJ mice (S3 Fig). These results indicate that *C*. *trachomatis* induces T_H_1 and T_H_17 responses in mice irrespective of the strain.

### T_H_1 immunity controlled *C*. *trachomatis* challenge infection

Because our ICS assays showed *Chlamydia*-specific CD4+ T cell effector function was primarily characterized by IFN-γ production, we posited T_H_1-type immunity conferred protection against ivag *C*. *trachomatis* challenge infection. To test this hypothesis, we compared *C*. *trachomatis* clearance of ivag challenge in mice at 60 dpi that were untreated or administered antibodies that blocked IFN-γ, IL-17, or IFN-γ and IL-17 signaling concomitant with challenge. Compared to untreated controls, clearance of *C*. *trachomatis* was marginally impaired in infected mice that received IL-17 blocking antibody, whereas bacterial burden was significantly larger and more persistent in mice after IFN-γ signaling blockade (with or without concurrent IL-17 blockade) (Fig 4C). Considered in conjunction with the ICS assay results for *Chlamydia*-specific CD4^+^ T cell effector function, these studies established that T_H_1 and T_H_17 immune responses form after primary ivag *C*. *trachomatis* infection, but that T_H_1 immunity primarily controls ivag challenge.

### T_H_17 immunity induced genital damage during *C*. *trachomatis* challenge infection

**Fig 5:**
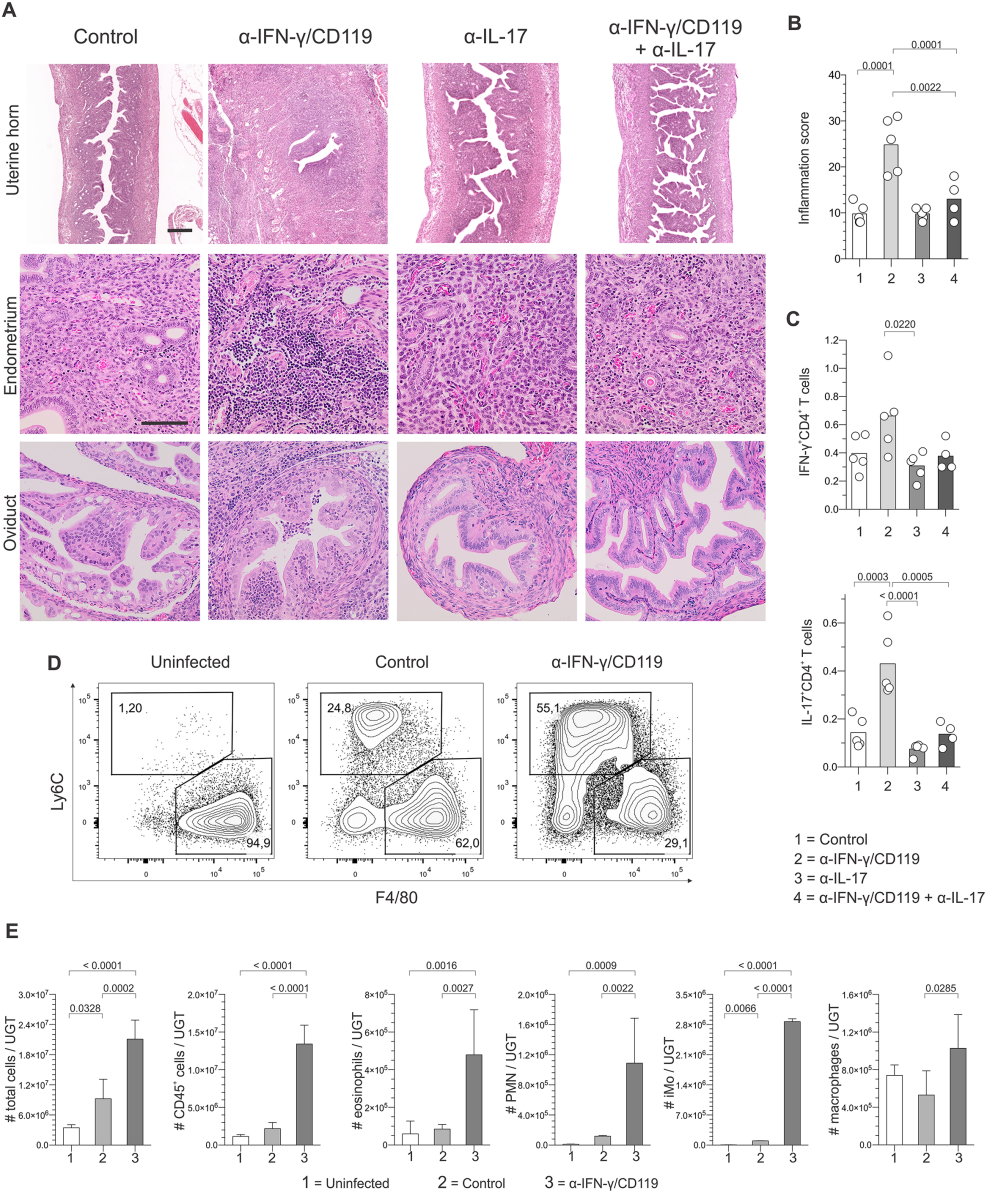
*Chlamydia*-specific T_H_17 immunity promoted genital tract damage. Groups of mice described in Fig 4C were euthanized at 21 dpc, and UGT processed for histological evaluation. (A) Representative results from H&E staining shows pervasive genital tract damage in mice treated with antibody blocking IFN-γ signaling characterized by UGT inflammation and intra-abdominal adhesions; (scale bar top row, 500 μm; scale bar lower rows, 50 μm). (B) Semi-quantitative scoring system identifies significant UGT inflammation in mice administered antibody blocking IFN-γ signaling. (C) In separate studies, DLN were excised at 5 dpc from controls or mice administered antibodies blocking IFN-γ, IL-17, or IFN-γ and IL-17 signaling concomitant with challenge, and processed into single-cell suspensions. Flow cytometry was used to quantify intracellular accumulation of IFN-γ and IL-17 by CD4^+^ T cells that responded to *Chlamydia* EB stimulation; (percentages of cytokine-producing CD4^+^ T cells are displayed) (bars indicate means). In other studies, flow cytometry was used to characterize UGT inflammation in uninfected controls, mice at 5 dpc, and mice at 5 dpc that were administered antibodies blocking IFN-γ signaling concomitant with the ivag challenge infection. (D) Representative contour plots for UGT macrophages and inflammatory monocytes displayed; (numbers denote percentage of the myeloid cell populations). (E) Heightened IL-17 secretion by CD4^+^ T cells induced by IFN-γ signaling blockade significantly increased numbers of eosinophils, inflammatory monocytes, polymorphonuclear neutrophils, and macrophages in the UGT at 5 dpc (values are mean ±SD). Results displayed in Fig 5 are representative of 2 independent experiments (n = 5 per group).

Blocking IFN-γ or IFN-γ and IL-17 comparably impaired genital clearance of ivag *C*. *trachomatis* challenge (Fig 4C). At 21 dpc however, we detected widespread genital tract inflammation and destruction only if mice received IFN-γ blocking antibody alone (Fig 5A and 5B, and S4 Fig). This implied *C*. *trachomatis*-specific T_H_17 immune responses in these animals were more important in promoting genital tract damage than the increased chlamydial burden. Consistent with this conclusion, *in vivo* IFN-γ blockade tripled the frequency of IL-17-producing *Chlamydia*-specific CD4^+^ T cells without significantly altering IFN-γ production (Fig 5C). IFN-γ blocking antibody administration also dramatically increased the numbers of polymorphonuclear neutrophils and inflammatory monocytes in the UGT at 5 dpc (Fig 5D and 5E). This T_H_17-mediated inflammation was likewise associated with prominent local and systemic changes, including extensive intra-abdominal adhesions and increased splenic weights (S4 Fig). This intense inflammation and tissue destruction created by IFN-γ signaling blockade was in particularly sharp contrast to the complete absence of UGT damage seen in wild type Balb/cJ mice after primary and challenge ivag infection with *C*. *trachomatis* infection serovar D (Figs 1 and 2). Analogous to IL-17-mediated UGT tissue damage observed in *C*. *muridarum*-infected mice during primary infection [27], our results thus revealed that *C*. *trachomatis*-specific T_H_17 immunity promotes immunopathological damage without significantly contributing to genital clearance of ivag challenge infection.

### Recurrent ivag *C*. *trachomatis* challenge infection reduced reproductive fertility

**Fig 6:**
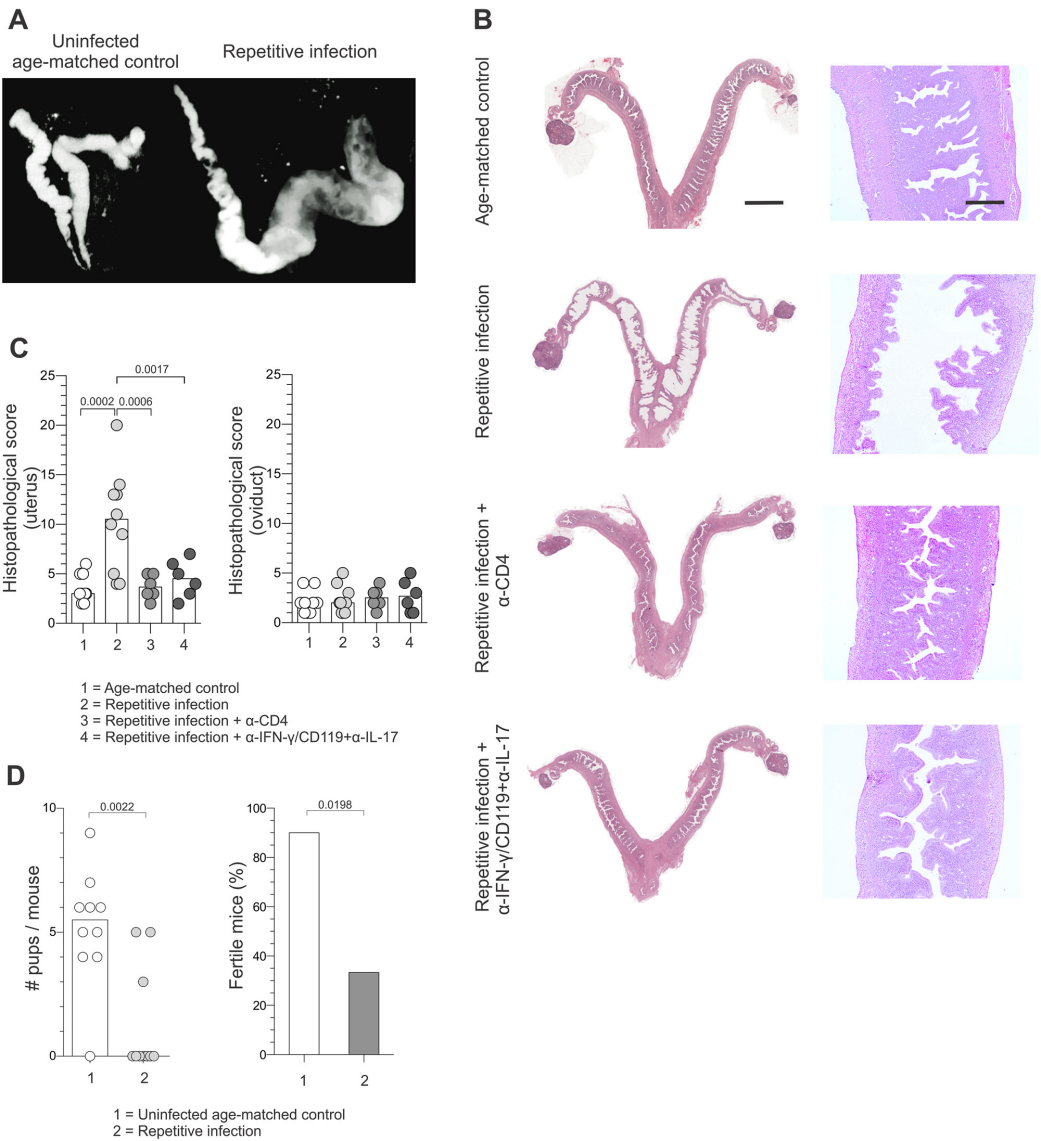
Recurrent ivag *C*. *trachomatis* challenge infection caused genital tract damage and reproductive infertility. Balb/cJ mice underwent primary ivag infection as described in Fig 1, and at 60 dpi were ivag challenged with 10^4^ IFU *C*. *trachomatis* serovar D 3 times per week for 3 weeks. (A) 21 days after the last challenge, water-soluble contrast medium was transcervically administered to infected mice and uninfected, age-matched controls to visualize the UGT with micro-CT imaging; (representative results shown, scale of 6 for both images). (B) In separate studies, *Chlamydia*-infected mice at 60 dpi were untreated or administered antibodies blocking IFN-γ and IL-17 signaling or depleting CD4^+^ cells during a similar 3 week course of repetitive ivag challenge infection with 10^4^ IFU of *C*. *trachomatis* serovar D. In addition, age-matched uninfected controls underwent this same 3 week course of infection. All mice were euthanized 21 days after completing the 3-week infection course to evaluate UGT pathology (representative H&E images shown) (scale bar left column, 4 mm; scale bar right column, 500 μm). (C) A semi-quantitative histopathological scoring system identifies the increased endometrial atrophy, cystic dilatation, and luminal distension in mice at 60 dpi that were repetitively challenged compared to repetitive challenge of previously uninfected mice or mice administered antibodies neutralizing IFN-γ and IL-17 signaling or depleting CD4^+^ cells throughout the 3 week course of challenge infections (bars denote means). (D) Compared to uninfected, age-matched controls, repetitive challenge significantly decreased fertility and number of pups born per female (n = 10 per group) (bars indicate medians or percentages) (results representative of 2 independent experiments).

As strong T_H_1 and T_H_17 responses were induced during a single ivag *C*. *trachomatis* challenge infection, we posited repetitive genital exposure to low infectious inoculums of the bacterium increases the risk of immunopathological damage. To test this hypothesis, *C*. *trachomatis*-infected mice at 60 dpi were ivag challenged with 10^4^ IFU of *C*. *trachomatis* 3 times per week for 3 weeks. Genital tracts were examined *in vivo* 21 days after completing the full 3-week course of repetitive infection using micro-CT imaging, and these images revealed that repeat infection induced cystic changes and profound uterine lumen distension not detected in uninfected, age-matched controls (Fig 6A, S1 and S2 Videos). Consistent with CT imaging results, macroscopic and histologic examination of mouse genital tracts after multiple ivag C. trachomatis challenge infections identified patent uterine dilation, hydrometra, marked widespread endometrial atrophy and some areas of stromal edema (Fig 6B and 6C, and S5 Fig). As anticipated by the CT images and histopathology data, repetitive infection with *C*. *trachomatis* serovar D significantly reduced the number of pups born per female and the fertility rate of these mice (Fig 6D). Likewise, repetitive infection with *C*. *trachomatis* serovar L2 elicited comparable UGT damage (S5 Fig). Importantly, the pronounced tissue remodeling produced by low-dose repetitive *C*. *trachomatis* infection (excluding the low-grade neutrophilic infiltrate) was not observed in mice administered antibodies that neutralized IFN-γ and IL-17 signaling or depleted CD4^+^ T cells concomitant with ivag challenge. Conversely, as shown before both these antibody treatments were found to impair chlamydial clearance. Our findings thus indicate that *Chlamydia*-specific immunity, and not increased bacterial burden, produced the widespread UGT damage observed after repetitive ivag infection.

## Discussion

Rare phenotypic disease expression in women with genital *C*. *trachomatis* infection makes it especially challenging to use experimental infections to model the typical course of clinical infection. It is similarly problematic for experimental infections to model human host responses to an exclusively human pathogen. In this investigation, we hoped to newly establish that mice infected with an oculogenital *C*. *trachomatis* serovar generate the robust T_H_2 immunity seen in *Chlamydia*-infected women. Initial evaluation of our infection model revealed that primary ivag *C*. *trachomatis* infection ascends into the UGT, but is eradicated without inducing obvious tissue damage. While this provided indication that T_H_2 immunity was potentially involved in the murine host response, T_H_1 and T_H_17 immunity were the host responses induced by *C*. *trachomatis* infection in our studies. In contrast, infection did not promote formation of *Chlamydia*-specific T_H_2 effector function. The predominately T_H_1 and T_H_17 immune responses we detected were therefore consonant with the responses reported in mouse genital infection models that infected with *C*. *muridarum* and *C*. *trachomatis* LGV serovars [20, 23, 24, 27].

Additional studies we performed in our mouse model of ivag infection with an oculogenital *C*. *trachomatis* serovar further revealed that T_H_1 immunity was primarily responsible for controlling ivag *C*. *trachomatis* challenge infection, and that T_H_17 immunity promoted immunopathological tissue damage. While novel, these findings are consistent with previous reports that indicated transfer of *Chlamydia*-specific CD4^+^ T cells protects against genital infection with *C*. *trachomatis* serovar L2 [20]. Though we found the course of infection more protracted in mice treated with antibodies depleting CD4^+^ T cells or blocking IFN-γ signaling, the 1-time ivag challenge infection in treated mice was eventually controlled. These results identify the presence, at least in mice, of IFN-γ-independent innate mechanisms capable of controlling genital *C*. *trachomatis* infection.

Our studies also newly characterized the inflammation induced by T_H_17 immune responses to *C*. *trachomatis* in the mouse female genital tract. T_H_17 immunity appeared to play a minor role in clearing this intracellular bacterial pathogen, but this response could elicited UGT tissue destruction. As negligible T_H_17 immunity is generated in women by genital *C*. *trachomatis* infection [12, 28], we speculate *Chlamydia*-specific T_H_17 memory responses were possibly evolutionarily selected against to prevent infection from reducing reproductive fitness. Similarly, our results indicate *Chlamydia*-specific T_H_1 immunity promotes bacterial clearance and immunopathological tissue destruction upon repetitive genital exposure to the bacterium. These findings are congruent with a nonhuman primate model of *Chlamydia* infection in which pathogen-specific T_H_1 responses to challenge infection triggered genital pathology [29]. However, these findings may have critical implications for *C*. *trachomatis* vaccine development, as substantial focus has been placed on developing vaccines that promote robust *Chlamydia*-specific T_H_1 memory [30–34]. Because any collateral tissue damage induced by *Chlamydia* vaccines promoting T_H_1 responses may develop gradually and only with repetitive genital exposure to the pathogen, it would be challenging to evaluate this effect in clinical trials. While such a possibility is mere speculation, our results do highlight the need for research to more thoroughly understand the *Chlamydia*-specific human host responses that protect against natural infection. Likewise, our results also highlight the need to evaluate candidate *C*. *trachomatis* vaccines in animal models that are more clinically relevant than the currently available mouse models.

In conclusion, our studies newly uncovered that the T_H_1 and T_H_17 immune responses elicited against *C*. *trachomatis* conferred exquisite protection against challenge infection while delivering devastating consequences for reproductive fertility. These findings provide novel illustration of an immune-driven defense strategy that reduces pathogen load but that is inappropriate for conserving host reproductive fitness. Conversely, tolerance is a strategy for host defense that minimally influences pathogen load but decreases the likelihood for disease expression in infected tissue [35–37]. As *C*. *trachomatis* infection of the human female genital tract rarely causes phenotypic disease expression, it is possible that tolerance, rather than an immune-driven resistance mechanism, is the human host defense evolutionarily selected to combat this pathogen. If correct, our findings imply that developing *C*. *trachomatis* vaccines that are both safe and effective requires full understanding of the mechanisms of disease tolerance in the human female genital tract that control genital infection without diminishing reproductive fitness.

## Materials and Methods

### Mice and infections

All animal experiments were approved by the Ohio State University Institutional Animal Care and Use Committee, and performed in accordance with the Guide for the Care and Use of Laboratory Animals. After primary or challenge ivag infection, members of our research team observed mice daily for clinical signs of illness (e.g., weight loss and inactivity). Although no mice became ill or died prior to a planned experimental endpoint, a protocol was in place to euthanize mice that became moribund or lost excessive weight. Once an experimental endpoint was achieved, mice were euthanized by carbon dioxide inhalation, and death confirmed by ascertaining cardiac and respiratory arrest. Where possible, measurements were acquired by investigators blinded to the assignment of mice to a particular experimental group.

For primary infection, 6–8 week-old female Balb/cJ, C.129S7(B6)-Ifng^tm1Ts^/J (IFN-γ^-/-^ Balb/cJ), or C57BL/6J mice (all strains obtained from Jackson Laboratories, Bar Harbor, ME) were injected subcutaneously (s.c.) with 1 mg depot-medroxyprogesterone acetate (DMPA) (Upjohn, Don Mills, Ontario, Canada). This pharmacologically relevant DMPA dose [38] ensured consistent infection, as susceptibility to genital *C*. *trachomatis* infection in the mouse is estrus cycle-dependent [16]. 5 days later, mice were sedated via intraperitoneal (i.p.) injection of 1.8 mg ketamine hydrochloride (Fort Dodge Animal Health, Fort Dodge, IA) and 0.18 mg xylazine (Lloyd Laboratories, Shenandoah, IA), and ivag infected once with 10^6^ IFU of the oculogenital strain UW-3/Cx of *C*. *trachomatis* serovar D (VR-885) (ATCC, Manassas, VA), or daily for 3 consecutive days with 10^4^ IFU of the oculogenital strain UW-3/Cx of *C*. *trachomatis* serovar D, the oculogenital strain BOUR of *C*. *trachomatis* serovar E (VR-348B), or the lymphogranuloma venereum (LGV II) strain 434 of *C*. *trachomatis* serovar L2 (VR-902B) (All from ATCC) in 10 μL of sucrose-phosphate-glutamate buffer (SPG). Previously described procedures [19] were followed for transcervical infection with 10^6^ IFU of *C*. *trachomatis* serovar D. For primary ivagl infection with mouse pneumonitis strain Nigg II of *C*. *muridarum* (VR-123) (ATCC), 2 x 10^5^ IFU were administered once in 10 μL of SPG. Bacterial clearance was determined by measuring *Chlamydia* DNA by RT-qPCR in CVL collected at indicated dpi. For 1-time challenge infection, mice at 60–90 dpi (in which RT-qPCR results from CVL collected at 30 dpi confirmed eradication of primary infection) were ivag infected with 10^6^ IFU of *C*. *trachomatis* serovar D in 10 μL of SPG. For repetitive challenge infections, mice at 60 dpi were ivag infected 3 times per week for 3 consecutive weeks with 10^4^ IFU of *C*. *trachomatis* serovar D or serovar L2 in 10 μL of SPG. Experimental interrogation of repetitively infected mice occurred 21 days after completing the entire course of infection. For single and repetitive challenges, 1 mg DMPA was administered 5 days before the initial challenge. Use of serial inoculations for primary infection and repetitive challenge were designed to model *C*. *trachomatis* sexual transmission, which is promoted by persistent infection and frequent contact in core groups of sexually active individuals [39]. Likewise, the infectious inoculum used for the repetitive infections was consistent with levels achieved in human biological specimens [22]. As it was possible the higher-dose infectious inoculums of *C*. *muridarum* and *C*. *trachomatis* used in prior research favored formation of T_H_1 and T_H_17 immunity, our study was specifically designed to learn if 10^4^ IFU of an oculogenital *C*. *trachomatis* serovar induced murine T_H_2-type responses comparable to those seen in women.

### *C*. *trachomatis* quantification

At indicated dpi or dpc, CVL or UGT were harvested to quantify *C*. *trachomatis* DNA levels by RT-qPCR. For CVL collection, 30 μL PBS were inserted into the vaginal vault and recovered. For UGT collection, mice were euthanized and whole UGT excised. The Clearview *Chlamydia* rapid test (Wampole Laboratories, Unipath, Bedford, UK) identiifed productive infection in real time (at 5 dpi) using CVL (all infected mice were positive in all experiments performed). Total DNA was isolated from samples with the DNeasy Blood & Tissue Kit (Qiagen, Valencia, CA) following manufacturer's instructions. Utilizing primers and methods previously described [40], *Chlamydia* (16S rRNA) and host (GAPDH) DNA were quantified in indicated extracted DNA samples utilizing a TaqMan assay-based RT-qPCR. IFU determinations were performed as previously described [41]. We also quantified *C*. *trachomatis* levels in CVL with the MicroTrak II Chlamydia EIA kit (Trinity Biotech PLC, Jamestown, NY) that detects *Chlamydia* LPS (a pure *C*. *trachomatis* EB standard with known IFU titer was used to convert absorbance data into IFU equivalents). For graphical representation of *Chlamydia* clearance data, non-detectable (ND) values were assigned the lowest value in the y-axis (i.e., for log scale presentation purposes), and indicated as such.

### Reagents and flow cytometric analysis

For ICS assays, iliac lymph nodes were excised at 5 dpc and processed into single-cell suspensions. Cells were re-suspended at a density of 10^6^ cells/ml in RPMI-1640 (Cellgro, Mediatech Inc, Manassas, VA) supplemented with 10% FBS (Atlanta Biologicals, Flowery Branch, GA), 2 mM L-glutamine, 1 mM sodium pyruvate, non-essential amino acids, 50 μM 2-ME, 100 U/ml penicillin, 100 μg/ml streptomycin, and 50 μg/ml gentamycin (termed complete media) (all Cellgro), and incubated for 36 h at 37°C in a 5% CO_2_ atmosphere with complete media alone or *C*. *trachomatis* serovar D EB (10^6^ IFU/ml) previously inactivated by γ-irradiation. For the final 6 h, cells were incubated with manufacturer’s recommended amounts of Brefeldin A (GolgiPlug^™^, BD Biosciences, San Diego, CA). Cells were stained with LIVE/DEAD^®^ Fixable Near-IR or Aqua Dead Cell stain (Invitrogen, Carlsbad, CA), incubated with anti-CD16/CD32 mAb (Fc Block^™^, BD Biosciences), and stained with various combinations of the following antibodies: FITC-conjugated anti-CD90.2 (53–2.1, BD Biosciences), AF700-conjugated anti-CD8α (53–6.7, BD Biosciences), PerCP-Cy5-5- conjugated anti-CD4 (RM4-5, eBioscience), BV510-conjugated anti-CD11b (BioLegend, San Diego, CA), PE-conjugated anti-CD4 (clone RM4-4, BioLegend), PerCP-Cy5.5-conjugated anti-CD45R (clone RA3-6B2, eBioscience, San Diego, CA), BV510-conjugated anti-CD90.2 (clone 53–2.1, BioLegend), and V450-conjugated anti-CD8 α (clone 53–6.7, BD Biosciences). After fixation and permeabilization with Cytofix/Cytoperm^™^ solution (BD Biosciences), cells were stained with combinations of the following antibodies: PE-conjugated anti-IL-17A (TC11-18H10, BD Biosciences), PE-Cy7-conjugated anti-TNF (MP6-XT22, BioLegend), APC-conjugated anti-IL-4 (11B11, BD Biosciences), eF450-conjugated anti-IFN-γ (XMG1.2, eBioscience), APC-conjugated anti-IL-17A (eBio17B7, eBioscience), AF488-conjugated anti-IFN-γ (XMG1.2, eBioscience), and PE-Cy7-conjugated anti-IL-4 (clone BVD6-24G2, eBioscience) diluted in Perm/Wash^™^ buffer (BD Biosciences). To characterize leukocyte subpopulations in *Chlamydia*-infected genital tissue, entire UGT were excised at 5 dpc and processed into single-cell suspensions using complete media containing 1mg/ml collagenase D (Roche, Indianapolis, IN), 1mg/ml Dispase II (Roche), and 0.25mg/ml DNase I (Sigma). Cell suspensions were placed on ice and 10nM EDTA (Lonza, Rockland, ME) added to stop collagenase D and Dispase II digestion. Cells were sequentially incubated with LIVE/DEAD^®^ Fixable Violet Dead Cell stain, Fc Block^™^, and stained with the following antibodies: FITC-conjugated anti-MHC-II (M5/114.15.2, eBioscience), PE-conjugated Ly6G (1A8, BD Biosciences), PE-CF596-conjugated anti-Siglec-F (E50-2440, BD Biosciences), PerCP-conjugated CD45 (30-F11, BD Biosciences), PE-Cy7-conjugated Ly6C (AL-21, BD Biosciences), APC-conjugated anti-CD115 (AFS98, eBioscience), BV510-conjugated CD11b (M1/70, BioLegend), and BV605-conjugated anti-F4/80 (BM8, BioLegend). To determine the efficacy of CD4^+^ and CD8^+^ T cell depletion during *C*. *trachomatis* infection, peripheral blood was collected from tail veins of individual mice into tubes containing heparin. Cells were stained with antibodies specific for CD4 (RM4-4, eBioscience) or CD8β (eBioH35-17.2, eBioscience) that recognize epitopes unaffected by binding of the depleting antibodies. Cells were collected on a LSR-II flow cytometer (BD Biosciences), and data analyzed using FlowJo software (Tree Star Inc, Ashland, OR). Fluorescence minus one controls were used to define gates that measured intracellular cytokine production by live CD4^+^ and CD8^+^ T cells and gates that defined myeloid populations of interest. Percentages of cytokine-producing T cell percentages were calculated by subtracting cytokine percentage values found in unstimulated samples (background).

### Cell depletion and cytokine neutralization

Where indicated, mice were injected i.p. with 100 μg of a CD4-depleting antibody (clone GK1.5), 100 μg of a CD8α-depleting antibody (clone 2.43), 200 μg of an IFN-γ blocking antibody (clone XMG1.2) concomitant with 200 μg of an IFN-γ receptor 1 (CD119) blocking antibody (clone GR-20), or 200 μg of an IL-17 blocking antibody (clone 17F3) (all antibodies from BioXCell, West Lebanon, NH). Injections were administered 1 day before challenge (i.e., for cell depletion) or concomitant (i.e., for cytokine neutralization) with challenge infection and then every other day until euthanasia. Depletion of individual T cell populations achieved by monoclonal antibody administration was routinely >98% (S6 Fig).

### Immunohistochemistry (IHC)

As indicated, excised genital tracts were paraffin-embedded, and immunohistochemically stained to identify CD45^+^ (clone 30-F11, BD Biosciences) or CD3ε^+^ (clone F7.2.38, Dako, Carpinteria, CA) cells. To quantify CD3ε^+^ cell infiltrates, 10 separate fields were photographed under 200x magnification, and CD3ε^+^ cells enumerated using ImageJ software [42]. Original images were converted to 8-bit black and white images, threshold levels adjusted to exclude non CD3ε^+^ cells, and number of individual CD3ε^+^ cells calculated using the analyze particles component of this software.

### Histopathology

Genital tracts were removed *en bloc* at indicated time points after ivag infection, challenge, or repetitive challenge, immediately preserved in buffered 4% formaldehyde solution, and embedded in paraffin. Paraffin-embedded, 5-μm sections containing cervix and both uterine horns and oviducts were stained with hematoxylin and eosin (H&E). Slides were scanned at 400x resolution by an Aperio high-throughput digital scanner (Leica Biosystems, Buffalo Grove, IL). These same genital tract structures were assessed independently for the presence of acute inflammation (neutrophilic infiltrates), chronic inflammation (lymphocytic infiltrates), stromal edema, fibrosis, and luminal distension with the following semi-quantitative histopathological aggregate scoring system: 0, no significant infiltration, edema, fibrosis or distension; 1, infiltration at a single focus, minimal edema, fibrosis or distension; 2, infiltration at two to four foci, mild edema, fibrosis or distension; 3, infiltration at more than four foci, moderate edema, fibrosis or distension; and 4, confluent infiltration, severe edema, fibrosis or distension. All scoring was performed blinded to infection status or treatment group, and component scores for each tissue were combined to provide aggregate scores. If inflammatory infiltrates alone were evaluated, only the acute and chronic inflammation scores were used in the aggregate scoring.

### X-ray micro-computed tomography (CT)

21 days after completing the entire course of repetitive ivag challenge infections, experimental mice and uninfected age-matched control mice were sedated and 10–30 μl of a diatrizoate meglumine and diatrizoate sodium solution (Gastrografin, Bracco Diagnostics Inc., Princeton, NJ) inserted into the uterus lumen as a single bolus using a NSET (ParaTechs Corporation, Lexington, KY). Mice were sedated for 30 minutes after administration of contrast medium to facilitate its uniform distribution prior to micro-CT (Inveon, Siemens AG, Munich, Germany) evaluation. Maintaining the same threshold in all scans, image data was reconstructed using Inveon acquisition workplace software. Results were analyzed using Inveon research workstation software.

### Fertility assessment

21 days after the entire course of repetitive ivag challenge infections was completed; fertility was evaluated in experimental mice and uninfected age-matched control mice by placing 3–4 control or experimental mice with a proven breeder male. Age-matched control mice received DMPA treatments in an identical manner as the experimental mice. Baseline weights were recorded, and weights evaluated daily. Mice were sacrificed if diagnosed pregnant (e.g., 3 days of consistent weight gain after timed male exposure), and embryo numbers recorded. If a mouse did not become pregnant after 28 d, she was housed with a different proven breeder male. If this mating was unproductive, the mouse was classified infertile, euthanized, and the genital tract excised for histologic analysis.

### Statistical considerations

Statistical analyses were performed using Prism 6 software (GraphPad, La Jolla, CA). Normality of the data was tested using the D’Agostino—Pearson omnibus K2 test, the Shapiro-Wilk test, or evaluation of the residuals depending on sample size in each group. Differences between 2 groups were compared by an unpaired Student t test with Welch’s correction or an unpaired Mann—Whitney U test, depending on data distribution. Fisher’s exact test was used to compare percentages of fertile mice. In *Chlamydia* clearance studies, the area under the curve (AUC) in the clearance curve for each mouse was calculated (total *Ct* DNA x dpi/dpc) and AUC values compared. To compare multiple groups, depending on data distribution, one-way ANOVA and Tukey’s multiple comparison post hoc test or the Kruskal—Wallis test on ranks and Dunn’s multiple comparison post hoc test were used (unless indicated, multiple comparisons were performed against each other group). P values < 0.05 were considered statistically significant.

## Supporting Information

S1 Fig Primary ivag *C*. *muridarum* infection of mice caused significant oviduct tissue damage. Uninfected Balb/cJ mice underwent primary genital infection with 1 or 3 doses of the indicated strains of *C*. *trachomatis* or *C*. *muridarum* or were not uninfected. Mice were euthanized at 90 dpi, and UGT tissue excised and processed for histopathological analysis. Semi-quantitative scoring systems for (A) uterine or (B) oviduct histopathology found no significant differences between uninfected age-matched controls (uninfected) and mice infected with *C*. *trachomatis*. Conversely, mice infected with a single dose of *C*. *muridarum* developed severe hydrosalpinx. Number and amount of infectious doses administered (low: 10^4^ IFU; high: 10^6^ IFU); route of infection (ivag; intrauterine (iu)), and strain of *Chlamydia* used (*C*. *trachomatis* serovar D, *Ct* D; serovar E, *Ct* E; serovar L2, *Ct* L2; *C*. *muridarum*, *Cm*) are indicated in each group’s label.
(PDF)

S2 Fig Hydrosalpinx formed in IFN-γ^-/-^ mice genitally infected with 10^4^ IFU *of C*. *trachomatis* serovar D. Wild type Balb/cJ mice and IFN-γ^-/-^ mice on a Balb/cJ background underwent primary genital infection with 10^4^ IFU of *C*. *trachomatis* serovar D as described in Fig 1. Mice were euthanized at 90 dpi, and UGT tissue excised and processed for histopathological analysis. (A) Representative microscopic images of the oviducts are shown (scale bar, 200 μm). (B) Semi-quantitative scoring for identification of uterine or oviduct histopathology.
(PDF)

S3 Fig C57BL/6J mice developed robust Type 1 *Chlamydia*-specific CD4^+^ and CD8^+^ T cell responses after genital *C*. *trachomatis* infection. At 60 days after primary ivag infection with *C*. *trachomatis* serovar D, C57BL/6J mice were ivag challenged with 10^6^ IFU of *C*. *trachomatis* serovar D. Mice were euthanized 5 days later, and DLN excised and processed into single-cell suspensions, and incubated with inactivated *Chlamydia* EB or media alone for flow cytometric analysis of intracellular cytokine accumulation. Percentages of cytokine-producing CD4^+^ and CD8^+^ T cells are displayed (n = 5) (bars indicate medians).
(PDF)

S4 Fig IFN-γ signaling blockade enhanced *Chlamydia*-specific T_H_17 immune responses inducing immunopathological genital tissue damage. (A) Representative macroscopic images of the UGT of individual mice from groups described in Fig 5A and 5B. Image from mouse administered antibody blocking IFN-γ signaling alone concomitant with challenges is characterized by extensive intra-abdominal adhesions, especially involving the right uterine horn, oviduct and ovary. No significant macroscopic findings were observed in other treatment groups, despite higher burden of *C*. *trachomatis* in mice receiving antibodies blocking IFN-γ and IL-17 signaling (Fig 4C). (B) Splenic weights from groups of mice described in Fig 5A and 5B showed the enhanced T_H_17 immunity stimulated by blockade of IFN-γ signaling was associated with significantly increased splenic weights.
(PDF)

S5 Fig Repetitive low-dose ivag challenge infections with *C*. *trachomatis* serovars D and L2 caused genital tissue damage. (A) Representative macroscopic images of the UGT of mice that underwent repetitive challenge infection with *C*. *trachomatis* serovar D and uninfected age-matched controls that underwent an identical course of repetitive infection as described in Fig 6B. Only image from mouse subjected to primary and challenge infection shows prominent bilateral uterine dilation. In separate experiments, Balb/cJ mice underwent primary ivag infection with *C*. *trachomatis* serovar L2 as indicated in S1 Fig or remained uninfected. 60 days later, both groups were ivag challenged with 10^4^ IFU of *C*. *trachomatis* serovar L2 (i.e., 3 times per week for 3 weeks). 21 days after challenges were completed, mice were euthanized and UGT tissue excised and processed for histopathological analysis. (B) Representative images of the uterine horns from mice in each group are displayed (scale bar, 200 μm). (C) Semi-quantitative scoring for uterine and oviduct histopathology.
(PDF)

S6 Fig Efficacy of CD4^+^ and CD8^+^ T cell depletion during *C*. *trachomatis* infection. Where indicated, Balb/cJ mice that underwent primary ivag *C*. *trachomatis* infection as described in Fig 1 were ivag challenged at 60–90 dpi with 10^6^ IFU of *C*. *trachomatis* serovar D. As specified, antibodies depleting CD4^+^ (clone GK1.5) or CD8^+^ (clone 2.43) T cells were administered 1 day prior to challenge, and then every other day until euthanasia. Representative contour plots show efficiency of CD4^+^ and CD8^+^ T cell depletions in peripheral blood specimens collected 2 days prior to euthanasia.
(PDF)

S1 Video Micro-CT image of an uninfected, age-matched female mouse. An uninfected, age-matched female Balb/cJ mouse, as indicated in Fig 6, was sedated for iu administration of Gastrografin via NSET. After 0.5 h, *in vivo* micro-CT imaging was performed (scale: 6).
(MP4)

S2 Video Micro-CT image of a mouse 3 weeks after completing the 3-week course of repetitive ivag challenge infections. As indicated in Fig 6, mouse was sedated for *in vivo* micro-CT imaging described in the caption for S1 Video (scale: 6).
(MP4)
