# Supplementary material for: Intravaginal Chlamydia trachomatis Challenge Infection Elicits TH1 and TH17 Immune Responses in Mice That Promote Pathogen Clearance and Genital Tract Damage
Source: PLoS One. 2016 Sep 8;11(9):e0162445. doi: 10.1371/journal.pone.0162445 (PMC5015975; doi:10.1371/journal.pone.0162445)
Supplement: S1 Fig — Uninfected Balb/cJ mice underwent primary genital infection with 1 or 3 doses of the indicated strains of C. trachomatis or C. muridarum or were not uninfected. Mice were euthanized at 90 dpi, and UGT tissue excised and processed for histopathological analysis. Semi-quantitative scoring systems for (A) uterine or (B) oviduct histopathology found no significant differences between uninfected age-matched controls (uninfected) and mice infected with C. trachomatis. Conversely, mice infected with a single dose of C. muridarum developed severe hydrosalpinx. Number and amount of infectious doses administered (low: 104 IFU; high: 106 IFU); route of infection (ivag; intrauterine (iu)), and strain of Chlamydia used (C. trachomatis serovar D, Ct D; serovar E, Ct E; serovar L2, Ct L2; C. muridarum, Cm) are indicated in each group’s label. (PDF) [file pone.0162445.s001.pdf]

Supporting information

Intravaginal Chlamydia trachomatis Challenge Infection Elicits T_H1 and T_H17 Immune Responses in Mice that Promote Pathogen Clearance and Genital Tract Damage

Rodolfo D. Vicetti Miguel*, Nirk E. Quispe Calla, Stephen D. Pavelko, Thomas L. Cherpes

*Corresponding author e-mail: vicettimiguel.1@osu.edu

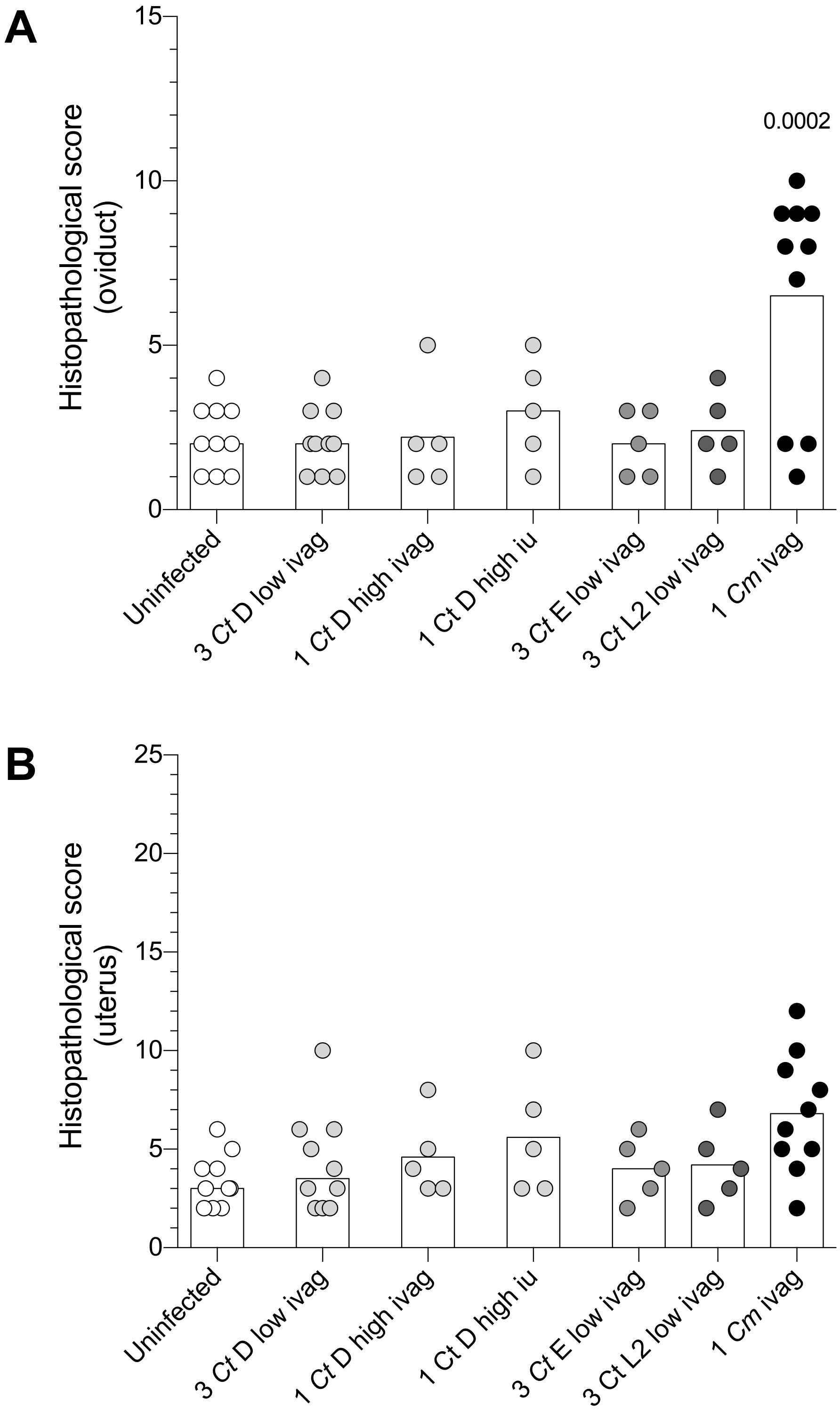

S1 Fig. Primary ivag *C. muridarum* infection of mice caused significant oviduct tissue damage.
