# Supplementary material for: Intravaginal Chlamydia trachomatis Challenge Infection Elicits TH1 and TH17 Immune Responses in Mice That Promote Pathogen Clearance and Genital Tract Damage
Source: PLoS One. 2016 Sep 8;11(9):e0162445. doi: 10.1371/journal.pone.0162445 (PMC5015975; doi:10.1371/journal.pone.0162445)
Supplement: S2 Fig — Wild type Balb/cJ mice and IFN-γ-/- mice on a Balb/cJ background underwent primary genital infection with 104 IFU of C. trachomatis serovar D as described in Fig 1. Mice were euthanized at 90 dpi, and UGT tissue excised and processed for histopathological analysis. (A) Representative microscopic images of the oviducts are shown (scale bar, 200 μm). (B) Semi-quantitative scoring for identification of uterine or oviduct histopathology. (PDF) [file pone.0162445.s002.pdf]

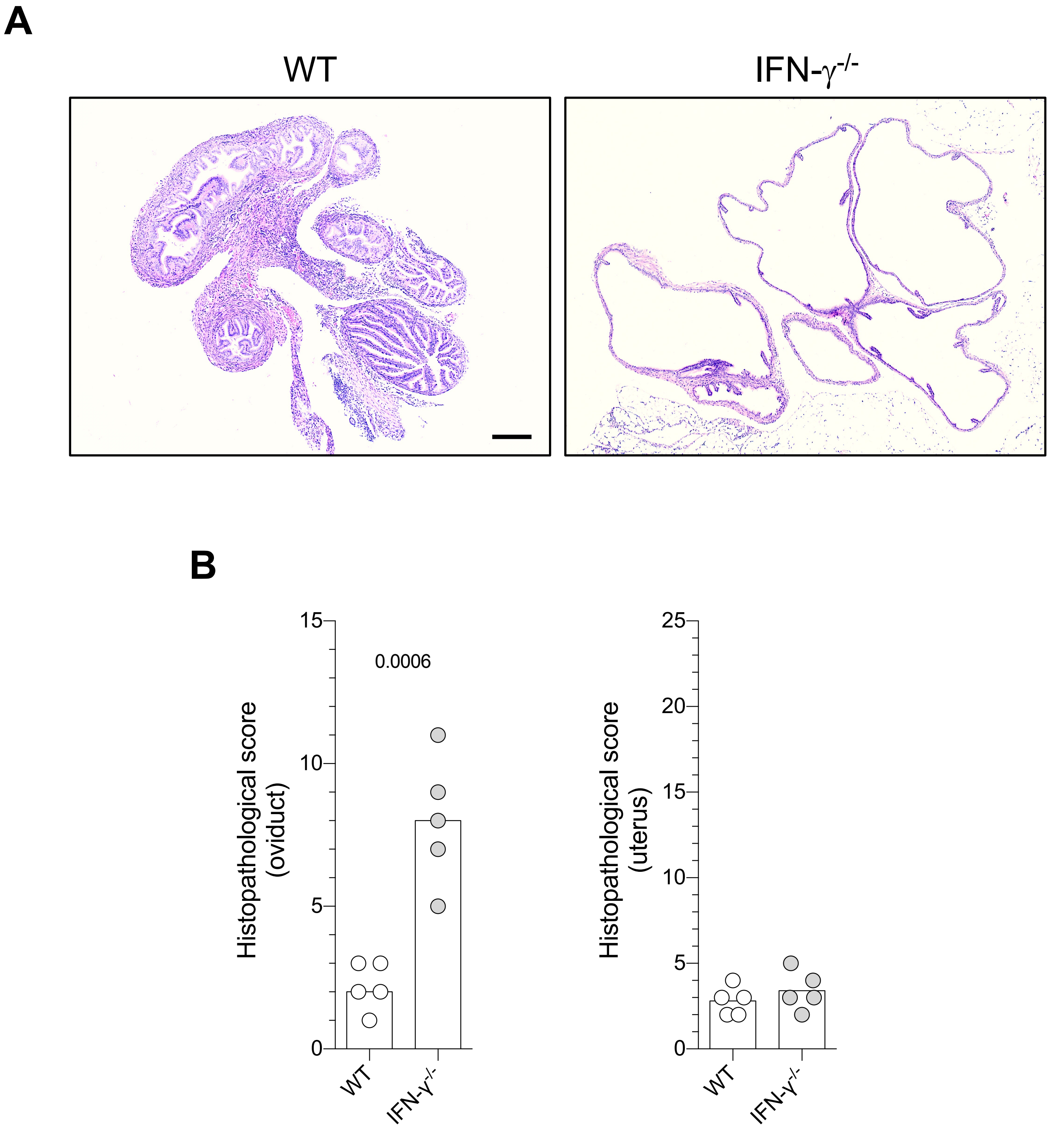

S2 Fig. Hydrosalpinx formed in IFN-γ$^{-/-}$ mice genitally infected with 10^4 IFU of *C. trachomatis* serovar D.
