# Supplementary material for: Intravaginal Chlamydia trachomatis Challenge Infection Elicits TH1 and TH17 Immune Responses in Mice That Promote Pathogen Clearance and Genital Tract Damage
Source: PLoS One. 2016 Sep 8;11(9):e0162445. doi: 10.1371/journal.pone.0162445 (PMC5015975; doi:10.1371/journal.pone.0162445)
Supplement: S3 Fig — At 60 days after primary ivag infection with C. trachomatis serovar D, C57BL/6J mice were ivag challenged with 106 IFU of C. trachomatis serovar D. Mice were euthanized 5 days later, and DLN excised and processed into single-cell suspensions, and incubated with inactivated Chlamydia EB or media alone for flow cytometric analysis of intracellular cytokine accumulation. Percentages of cytokine-producing CD4+ and CD8+ T cells are displayed (n = 5) (bars indicate medians). (PDF) [file pone.0162445.s003.pdf]

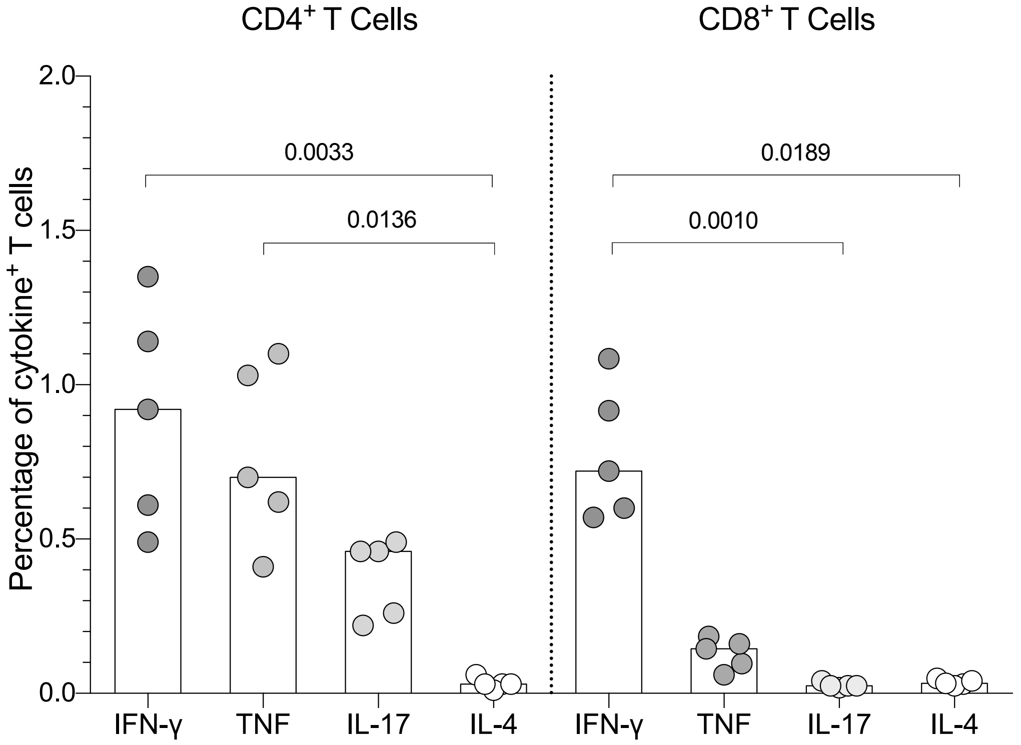

S3 Fig. C57BL/6J mice developed robust Type 1 *Chlamydia*-specific $CD4^+$ and $CD8^+$ T cell responses after genital *C. trachomatis* infection.
