# Supplementary material for: Intravaginal Chlamydia trachomatis Challenge Infection Elicits TH1 and TH17 Immune Responses in Mice That Promote Pathogen Clearance and Genital Tract Damage
Source: PLoS One. 2016 Sep 8;11(9):e0162445. doi: 10.1371/journal.pone.0162445 (PMC5015975; doi:10.1371/journal.pone.0162445)
Supplement: S4 Fig — (A) Representative macroscopic images of the UGT of individual mice from groups described in Fig 5A and 5B. Image from mouse administered antibody blocking IFN-γ signaling alone concomitant with challenges is characterized by extensive intra-abdominal adhesions, especially involving the right uterine horn, oviduct and ovary. No significant macroscopic findings were observed in other treatment groups, despite higher burden of C. trachomatis in mice receiving antibodies blocking IFN-γ and IL-17 signaling (Fig 4C). (B) Splenic weights from groups of mice described in Fig 5A and 5B showed the enhanced TH17 immunity stimulated by blockade of IFN-γ signaling was associated with significantly increased splenic weights. (PDF) [file pone.0162445.s004.pdf]

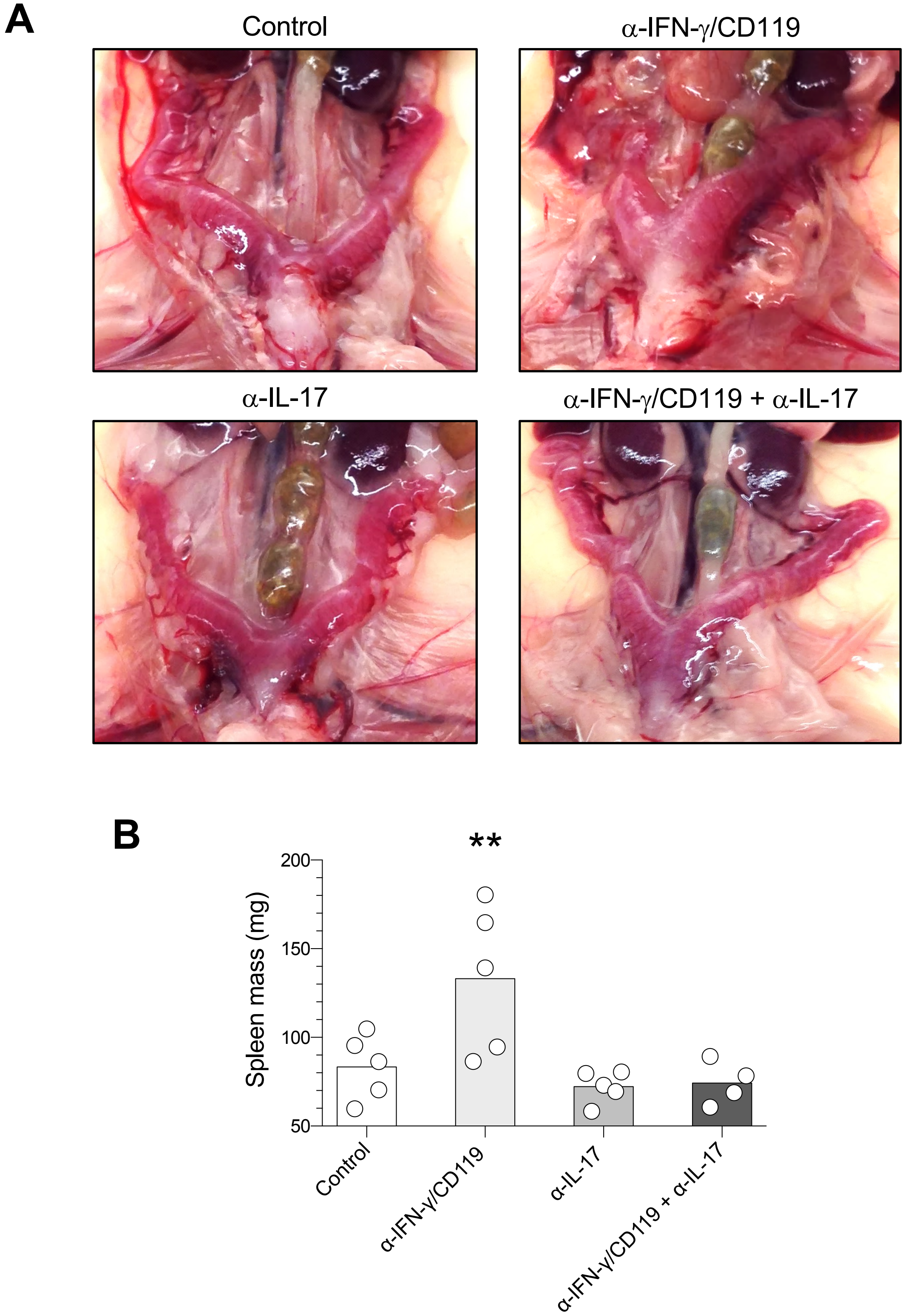

S4 Fig. IFN-γ signaling blockade enhanced *Chlamydia*-specific T_H17 immune responses inducing immunopathological genital tissue damage.
