# Supplementary material for: Intravaginal Chlamydia trachomatis Challenge Infection Elicits TH1 and TH17 Immune Responses in Mice That Promote Pathogen Clearance and Genital Tract Damage
Source: PLoS One. 2016 Sep 8;11(9):e0162445. doi: 10.1371/journal.pone.0162445 (PMC5015975; doi:10.1371/journal.pone.0162445)
Supplement: S5 Fig — (A) Representative macroscopic images of the UGT of mice that underwent repetitive challenge infection with C. trachomatis serovar D and uninfected age-matched controls that underwent an identical course of repetitive infection as described in Fig 6B. Only image from mouse subjected to primary and challenge infection shows prominent bilateral uterine dilation. In separate experiments, Balb/cJ mice underwent primary ivag infection with C. trachomatis serovar L2 as indicated in S1 Fig or remained uninfected. 60 days later, both groups were ivag challenged with 104 IFU of C. trachomatis serovar L2 (i.e., 3 times per week for 3 weeks). 21 days after challenges were completed, mice were euthanized and UGT tissue excised and processed for histopathological analysis. (B) Representative images of the uterine horns from mice in each group are displayed (scale bar, 200 μm). (C) Semi-quantitative scoring for uterine and oviduct histopathology. (PDF) [file pone.0162445.s005.pdf]

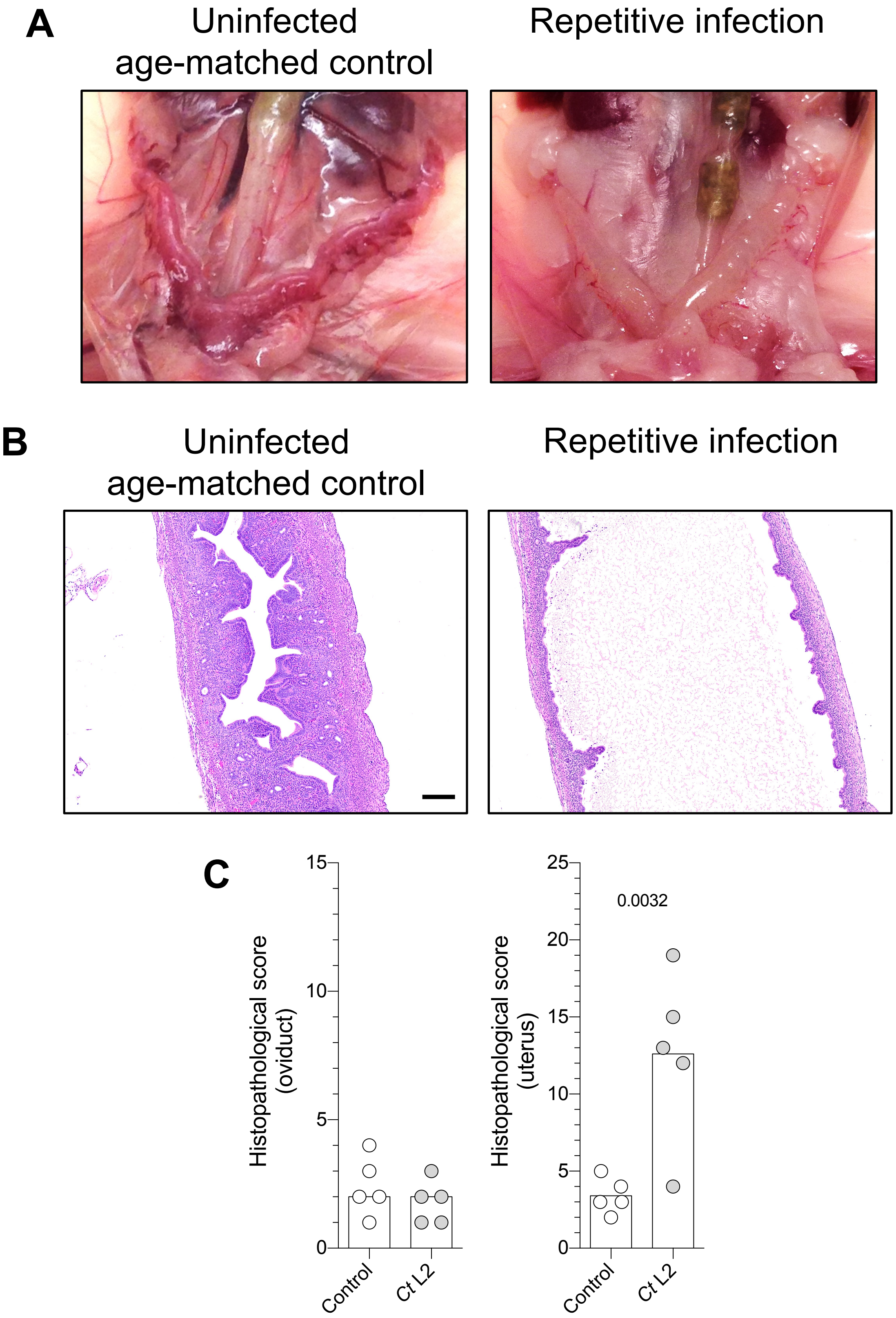

S5 Fig. Repetitive low-dose ivag challenge infections with *C. trachomatis* serovars D and L2 caused genital tissue damage.
