# Supplementary figures and images for: Intravaginal Chlamydia trachomatis Challenge Infection Elicits TH1 and TH17 Immune Responses in Mice That Promote Pathogen Clearance and Genital Tract Damage
Source: PLoS One. 2016 Sep 8;11(9):e0162445. doi: 10.1371/journal.pone.0162445 (PMC5015975; doi:10.1371/journal.pone.0162445)

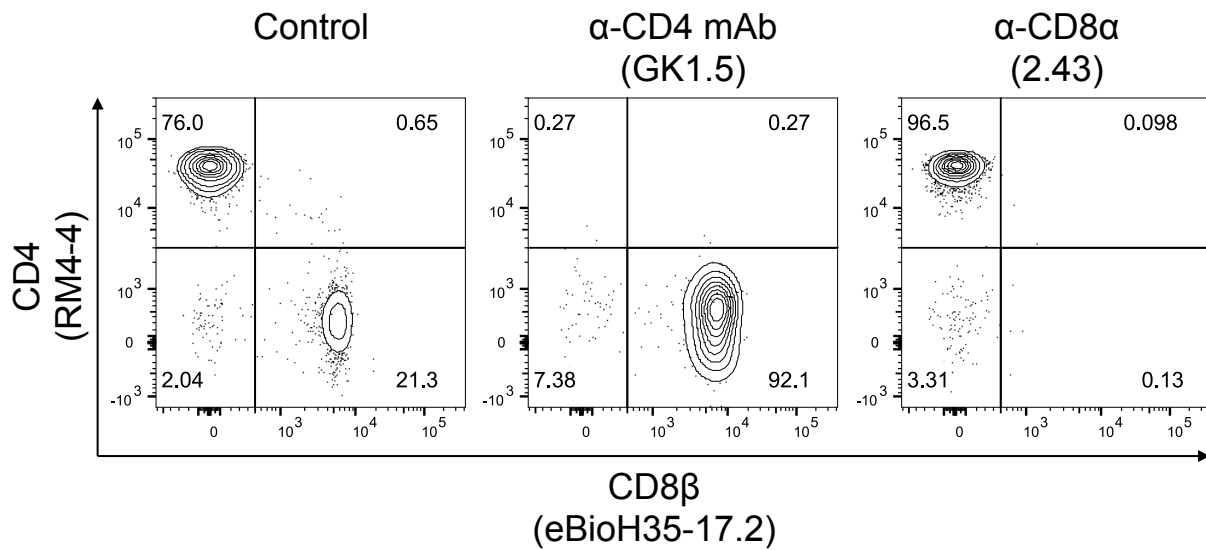

**S6 Fig.** Efficacy of CD4<sup>+</sup> and CD8<sup>+</sup> T cell depletion during *C. trachomatis* infection.

Supplement: S6 Fig — Where indicated, Balb/cJ mice that underwent primary ivag C. trachomatis infection as described in Fig 1 were ivag challenged at 60–90 dpi with 106 IFU of C. trachomatis serovar D. As specified, antibodies depleting CD4+ (clone GK1.5) or CD8+ (clone 2.43) T cells were administered 1 day prior to challenge, and then every other day until euthanasia. Representative contour plots show efficiency of CD4+ and CD8+ T cell depletions in peripheral blood specimens collected 2 days prior to euthanasia. (PDF) [file pone.0162445.s006.pdf]
